# Finite set model predictive control of permanent magnet synchronous motor current based on super twisting sliding mode observer

**DOI:** 10.1371/journal.pone.0336702

**Published:** 2025-12-01

**Authors:** Huanhuan Ren, Chengzhi Su, Ranxiang Long

**Affiliations:** College of Mechanical and Electric Engineering, Changchun University of Science and Technology, Changchun, China; University of Shanghai for Science and Technology, CHINA

## Abstract

This paper proposes a current model predictive control strategy for the permanent magnet synchronous motor (PMSM) based on a novel sliding mode observer to reduce the cost of PMSM and ensure good tracking performance. A super twisting sliding mode observer (STSMO) is designed to address the issues of high-frequency chattering and noise sensitivity caused by the large positive gain of traditional SMO. The discontinuous effect of the traditional SMO switching function is introduced into the derivative of the control rate, and a smooth estimate of the back electromotive force (EMF) is obtained through integration. Replace the sign function with a sigmoid function with smooth continuity to further reduce the chattering effect. To enhance the dynamic performance of the PMSM current loop, a finite control set model predictive control (FCS-MPC) strategy is employed in place of the conventional PI controller. Within each sampling period, all possible switching states are evaluated, and the optimal one is selected and directly applied to the inverter. Additionally, a dual-vector model predictive current control (DVMPCC) method is adopted to reduce current ripple. This approach synthesizes a voltage vector with arbitrary magnitude and direction by combining two voltage vectors within each sampling period. Numerical results demonstrate that the proposed sensorless PMSM predictive current control method achieves high accuracy in speed estimation and excellent dynamic response performance.

## Introduction

The rapid advancement of industrial technology has heightened the importance of high-performance motor systems. PMSM are widely used in electric vehicles and robotics due to their high efficiency, power density, and controllability [1,2]. However, PMSM control system design poses significant challenges from nonlinear dynamics, parameter variations, and external disturbances, demanding sophisticated control strategies for effective mitigation [3].

The high-performance drive control strategy for permanent magnet synchronous motors (PMSMs) relies fundamentally on precise rotor position feedback. In pursuit of cost reduction and space savings, numerous industrial enterprises and research institutions have dedicated significant efforts to developing sensorless control technologies, particularly focusing on model reference adaptive systems [4,5], Kalman filtering method [6,7], and sliding mode observer [8]. SMO has proven to be a robust state estimation method that can effectively determine motor state variables. However, due to its discontinuous control law, traditional SMO implementations exhibit inherent chattering that adversely affects speed estimation accuracy. In Reference [9], the authors introduced continuous functions to replace switching functions and designed a fuzzy sliding mode observer that adjusts the parameters of these continuous functions in real-time through fuzzy rules, thereby achieving smoother extraction of back EMF signals. To address the issues of chattering and significant observation errors in traditional SMO-based sensorless control of PMSM, [10] proposed a composite reaching law algorithm combining exponential reaching law and sinusoidal saturation function approaches. This improved sliding mode observer achieved an 80% reduction in speed estimation error. In Reference [11], a novel sliding mode control (SMC) strategy was developed for PMSM. This strategy incorporates an adaptive super-twisting algorithm to effectively mitigate the chattering phenomenon while enhancing the capability to suppress external disturbances. Initially, a sliding surface is constructed based on the dynamic model of the PMSM and real-time feedback. The super-twisting algorithm is then adaptively applied to dynamically adjust the control effort required to maintain the sliding mode. This ensures precise and timely intervention, thereby guaranteeing system stability and improving response speed. In [12], a new adaptive hybrid exponential convergence law was proposed to enhance the sliding mode control SMC system of PMSM. This method combines adaptive exponential components to achieve fast convergence with minimal overshoot, and is supplemented by a high gain interference observer for effective interference compensation. Regarding the reduction of sliding mode buffeting, [13] designed an adaptive fractional order sliding mode controller based on a fractional order sliding mode disturbance observer, which uses a new sliding mode approximation law instead of the traditional exponential approximation law to reduce system jitter and improve the control accuracy of the system. It should be noted that processing high-frequency discontinuous switching signals with low-pass filters introduces both amplitude attenuation and phase lag into the extended back EMF estimates. However, compensation for these effects is necessary when using either arctangent functions or a phase-locked loop (PLL) for speed and rotor position estimation [14–16].

Meanwhile, model predictive control (MPC) has garnered significant attention for its explicit capability to handle system constraints and predict future states, leading to its widespread application in current, speed, and position control of PMSMs [17–19]. In PMSM applications, MPC can directly incorporate control objectives into the cost function according to different control requirements. Based on whether PWM modulation is required – that is, depending on the discrete characteristics of the converter – MPC can be categorized into continuous control set MPC (CCS-MPC) and finite control set MPC (FCS-MPC). CCS-MPC [20,21] employs mathematical tools to optimize the constructed cost function, obtaining optimal control variables that are then applied to the control object through PWM. This approach features fixed switching frequency but requires substantial computational effort. In contrast, FCS-MPC [22,23] explicitly considers the discrete switching characteristics of the converter in its prediction model. It evaluates all possible switching combinations to predict the system state at the next time instant, and selects the voltage vector that minimizes the cost function as the optimal control action to be directly applied. This method offers fast dynamic response but faces challenges in multi-step prediction. Early research by [24,25] investigated the application of MPC in AC drive systems using FCS-MPC, establishing the foundation for widespread adoption of finite-set predictive control in AC motor drive systems with various converter topologies, including two-level inverters, multilevel inverters, and matrix converters.

A comparative study in Reference [26] implemented both FCS-MPC and CCS-MPC schemes for predictive torque control in PMSMs. The FCS-MPC approach was designed to select the optimal modulation intervals and output voltage vectors directly. In contrast, the CCS-MPC strategy formulated a predictive control structure for current and torque, integrated with a predictive current controller. Experimental comparisons revealed that both methods deliver satisfactory performance, with CCS-MPC achieving lower torque and current ripple, while FCS-MPC provided a faster torque response. Motivated by these findings, subsequent research has focused on numerous enhancements to further improve MPC performance [18,27,28].

This paper proposes a model predictive current control (MPCC) strategy for PMSM, utilizing a super-twisting sliding mode observer. The approach synergistically integrates the strengths of sliding mode observation and model predictive control to enhance the overall performance of PMSM drive systems. As an advanced form of SMO, the STSMO provides improved estimation accuracy and faster convergence, making it well-suited for the demands of PMSM control. Meanwhile, the MPCC scheme achieves optimal performance under system constraints by predicting and optimizing future current behavior. The paper is structured as follows: Section 2 introduces the mathematical model of the PMSM drive system. The design of the super-twisting sliding mode observer and the model predictive current controller are detailed in Sections 3 and 4, respectively. Section 5 presents a comparative analysis of simulation results under various operating conditions. Finally, Section 6 concludes the paper with key findings and implications.

### Design principles and processes

#### PMSM mathematical model.

**Fig 1:**
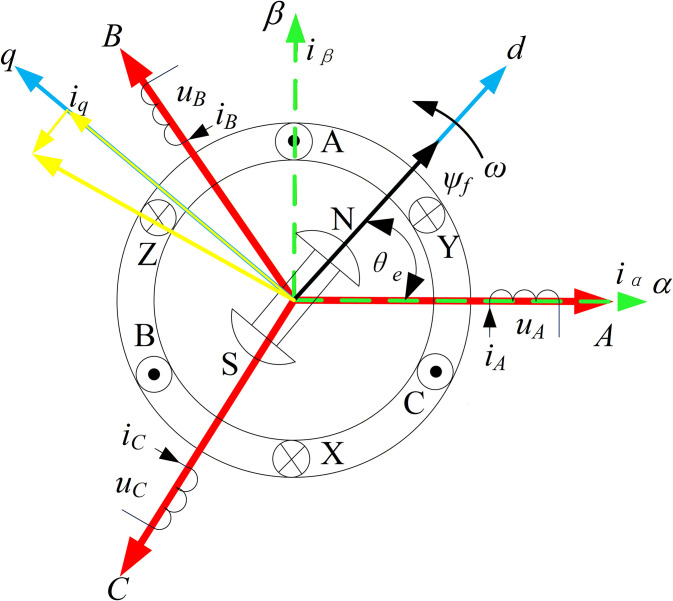
Vector transformation coordinate systems of PMSM.

Due to its complex structure, even when neglecting minor nonlinear effects, the mathematical model of a PMSM still exhibits high-order, multivariable, time-varying, and strongly coupled characteristics, making it difficult to directly solve its differential equations. By employing coordinate transformations (Clarke transform and Park transform), the complex physical model of the PMSM in the three-phase stationary coordinate system {ABC} can be equivalently converted into a DC motor-like model in the synchronous rotating two-phase coordinate system {dq}. This achieves linear decoupling of the model and enables vector control, as illustrated in Fig 1.

By transforming into the synchronous reference frame {dq}, the dynamic model of the PMSM can be derived. In this coordinate system, the voltage equations and flux linkage equations of the PMSM are expressed as (1) and (2), respectively:


$$\left\{ \begin{array}{c} u_{d}=\frac{d\psi_{d}}{dt}-\omega_{e}\psi_{q}+Ri_{d}\\ u_{q}=\frac{d\psi_{q}}{dt}+\omega_{e}\psi_{d}+Ri_{q} \end{array}\right.$$ (1)

$$\left\{ \begin{array}{c} \psi_{d}=L_{d}i_{d}+\psi_{f}\\ \psi_{q}=L_{q}i_{q} \end{array}\right.$$ (2)

where $u_{d}$ and $u_{q}$ represent the d axis and q axis voltages, respectively; $i_{d}$ and $i_{q}$ denote the d axis and q axis currents; $\psi_{d}$ and $\psi_{q}$ stand for the d axis and q axis flux linkages; R is the equivalent stator resistance; $L_{d}$ and $L_{q}$ are the d axis and q axis inductances of the stator windings; $\psi_{f}$ represents the permanent magnet flux linkage.

Generally, (1) is used for PMSM vector control. Currently, most SMO algorithms are designed based on the mathematical model in the stationary reference frame {αβ}, because angular velocity and position information can be easily extracted in this reference frame. The model can then be expressed as follows:


$$\left[\begin{array}{c} u_{\alpha }\\ u_{\beta } \end{array}\right]=\left[\begin{array}{cc} {}*20cR+\frac{d}{dt}\;\;L_{d} & \omega_{e}(L_{d}-L_{q})\\ -\omega_{e}(L_{d}-L_{q}) & R+\frac{d}{dt}L_{d} \end{array}\right]\left[\begin{array}{c} i_{\alpha }\\ i_{\beta } \end{array}\right]+\left[\begin{array}{c} e_{\alpha }\\ e_{\beta } \end{array}\right]$$ (3)

where $u_{\alpha }$, $u_{\beta }$, $i_{\alpha }$, $i_{\beta }$ and $e_{\alpha }$, $e_{\beta }$ represent the stator voltages, stator currents, and extended back EMF (electromotive force) in the two-phase stationary coordinate system, respectively, which satisfy:


$$\left[\begin{array}{c} e_{\alpha }\\ e_{\beta } \end{array}\right]=\left[(L_{d}-L_{q})(\omega_{e}i_{d}-\frac{di_{q}}{d_{t}})+\omega_{e}\psi_{f}\right]\left[\begin{array}{c} -\sin\theta_{e}\\ \;\;\cos\theta_{e} \end{array}\right]$$ (4)

From (4), it can be observed that the back-EMF signal contains information about both rotor speed and position. Therefore, after estimating the back EMF signal using an observer, the rotor speed and position can be obtained. To facilitate the application of a SMO for estimating the extended back-EMF, equation (3) can be rewritten in the form of a current state equation as follows:


$$\frac{d}{dt}\left[\begin{array}{c} i_{\alpha }\\ i_{\beta } \end{array}\right]=A\left[\begin{array}{c} i_{\alpha }\\ i_{\beta } \end{array}\right]+\frac{\text{1}}{L_{d}}\left[\begin{array}{c} u_{\alpha }\\ u_{\beta } \end{array}\right]-\frac{\text{1}}{L_{d}}\left[\begin{array}{c} e_{\alpha }\\ e_{\beta } \end{array}\right]$$ (5)

where $A=\frac{\text{1}}{L_{d}}\left[\begin{array}{cc} {}*20c-R & -\omega_{e}\left(L_{d}-L_{q}\right)\\ \omega_{e}\left(L_{d}-L_{q}\right) & -R \end{array}\right]$, $i_{\alpha }$ and $i_{\beta }$ are measurable (calculated) current output values, $u_{\alpha }$ and $u_{\beta }$ are he motor input voltages, $\omega_{e}$ and $\theta_{e}$ denote the unmeasurable rotor electrical angular velocity and electrical angle, respectively.

#### Traditional sliding mode observer.

(5) shows that both $e_{\alpha }$ and $e_{\beta }$ can be theoretically calculated directly from the current equations. However, in practical applications, analytical methods often yield inaccurate results due to motor parameter variations, measurement errors, and disturbances. The SMO can effectively overcome these issues. To estimate the back EMF, the traditional SMO is typically designed as follows:


$$\frac{d}{dt}\left[\begin{array}{c} {\hat{i}}_{\alpha }\\ {\hat{i}}_{\beta } \end{array}\right]=A\left[\begin{array}{c} {\hat{i}}_{\alpha }\\ {\hat{i}}_{\beta } \end{array}\right]+\frac{\text{1}}{L_{d}}\left[\begin{array}{c} u_{\alpha }\\ u_{\beta } \end{array}\right]-\frac{\text{1}}{L_{d}}\left[\begin{array}{c} {\hat{e}}_{\alpha }\\ {\hat{e}}_{\beta } \end{array}\right]$$ (6)

where superscript (∘) indicates an observed value.

Subtracting (5) from (6) yields the equation for current observation error:


$$\frac{d}{dt}\left[\begin{array}{c} {\bar{i}}_{\alpha }\\ {\bar{i}}_{\beta } \end{array}\right]=A\left[\begin{array}{c} {\bar{i}}_{\alpha }\\ {\bar{i}}_{\beta } \end{array}\right]+\frac{\text{1}}{L_{d}}\left[\begin{array}{c} e_{\alpha }-{\hat{e}}_{\alpha }\\ e_{\beta }-{\hat{e}}_{\beta } \end{array}\right]$$ (7)

where ${\bar{i}}_{\alpha }={\hat{i}}_{\alpha }-i_{\alpha }$ and ${\bar{i}}_{\beta }={\hat{i}}_{\beta }-i_{\beta }$ are current observation errors; ${\hat{e}}_{\alpha }$ and ${\hat{e}}_{\beta }$ are the observed back EMF, which can be represented by (8):


$$\left[\begin{array}{c} {\hat{e}}_{\alpha }\\ {\hat{e}}_{\beta } \end{array}\right]=\left[\begin{array}{c} h\cdot sign({\hat{i}}_{\alpha }-i_{\alpha })\\ h\cdot sign({\hat{i}}_{\beta }-i_{\beta }) \end{array}\right]$$ (8)

where h represents the sliding mode gain coefficient. To ensure observer convergence, h must satisfy (9):


$$h>\max\left\{\begin{array}{c} -R\left|{\bar{i}}_{\alpha }\right|+e_{\alpha }\cdot sign\left({\bar{i}}_{\alpha }\right)-\omega_{e}\left(L_{d}-L_{q}\right){\bar{i}}_{\beta }\cdot s\mathrm{\backslash nolimits}ign({\bar{i}}_{\alpha })\\ -R\left|{\bar{i}}_{\beta }\right|+e_{\beta }\cdot sign\left({\bar{i}}_{\beta }\right)-\omega_{e}\left(L_{d}-L_{q}\right){\bar{i}}_{\alpha }\cdot s\mathrm{\backslash nolimits}ign({\bar{i}}_{\beta }) \end{array}\right\}$$ (9)

When the observer’s state variables reach the sliding surfaces ${\bar{i}}_{\alpha }=0$ and ${\bar{i}}_{\beta }=0$, the observer states will remain on these surfaces thereafter. The sign function (signum), which outputs +1 for positive inputs and −1 for negative values, induces high-frequency switching in the actual control signal. To obtain continuous estimates of the extended back EMF, a low-pass filter (LPF) must be incorporated. However, the LPF introduces both amplitude attenuation and phase delay in the back EMF estimates, necessitating compensation for the rotor position estimation. The electrical position can be extracted from the estimated back EMF components using either an arctangent function or a phase-locked loop (PLL) circuit. This study employs the former approach, while PLL design methodologies can be found in references [29,30]:


$${\hat{\theta }}_{e}={\hat{\theta }}_{LPF}+\arctan\frac{{\hat{\omega }}_{e}}{\omega_{c}}$$ (10)

where θ^LPF=−arctan(e^α_LPF/e^α_LPFe^β_LPF\nulldelimiterspacee^β_LPF), $\omega_{c}$ represents the cutoff frequency of the low-pass filter.

By performing differentiation on 10, speed information can be obtained. Specifically, for surface-mounted permanent magnet synchronous motors, the speed estimate can be calculated as:


$${\hat{\omega }}_{e}={\left(1+\frac{{\hat{\omega }}_{e}^{2}}{\omega_{c}^{2}}\right)}^{\frac{\text{1}}{\text{2}}}\frac{\sqrt{e_{\alpha \_LPF}^{2}+e_{\beta \_LPF}^{2}}}{\psi_{f}}$$ (11)

**Fig 2:**
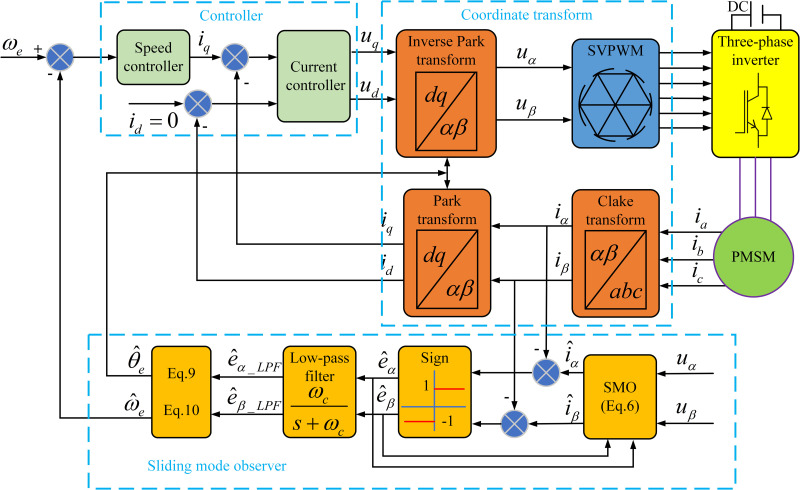
A block diagram of sensorless field oriented control with SMO.

The speed estimation method presented in (11) explicitly compensates for the amplitude attenuation of the back EMF induced by the low-pass filter, which is often overlooked in other common studies. The implementation framework of sensorless vector control for permanent magnet synchronous motor and the structure of traditional SMO algorithm are shown in Fig 2.

#### Super twisting sliding mode observer.

The SMO-based sensorless control algorithm offers simplicity and strong robustness, but cannot eliminate the chattering problem caused by high-frequency switching near the sliding surface, necessitating the introduction of a LPF for filtering and compensation. To mitigate chattering effects, this paper proposes incorporating the super twisting algorithm (STA) to design a super twisting sliding mode observer (STSMO). The stability and finite-time convergence of this approach have been proven in reference [31]. The basic form of the STA with perturbation is designed as:


{x˙1=−k1|x¯1|1/12\nulldelimiterspace2sign(x¯1)+x2+ρ1(x1,t)x˙2=−k2sign(x¯1)+ρ2(x1,t) (12)

where $x_{i}$ represents the system state variable, $k_{i}$ denotes the sliding mode coefficient, ${\bar{x}}_{i}$ corresponds to the error between estimated and actual state values, and $\rho_{i}$ signifies the disturbance term.

It has been proved in [24] that if the perturbation terms in(12) are globally bounded by


|ρ1|≤δ1|x1|1/12\nulldelimiterspace2,ρ2=0 (13)

and the gains $k_{1}$, $k_{2}$ satisfy:


$$k_{1}>2\delta_{1},k_{2}>k_{1}\frac{5\delta_{1}k_{1}+4_{\delta }^{12}}{2\left(k_{1}-2\delta_{1}\right)}$$ (14)

then the system will converge in finite time to sliding surface, where $\delta_{1}$ is any positive constant. x=0 is a globally asymptotically stable equilibrium point, and the system will converge to this equilibrium in finite time from any initial state.

To estimate the rotor position using the α and β current estimate as the state variables, this paper proposes an STSMO-based stator current observer for sensorless control of surface-mounted PMSM ($L_{d}=L_{q}=L_{s}$), constructed as follows:


ddt[i^αi^β]=[1Ls(−k1|i¯α|1/12\nulldelimiterspace2sign(i¯α)−∫k2sign(i¯α)dt)+ρ1(i^α,t)1Ls(−k1|i¯β|1/12\nulldelimiterspace2sign(i¯β)−∫k2sign(i¯β)dt)+ρ2(i^β,t)] (15)

Comparing (12) and (6), perturbation terms $\rho_{1}\left({\hat{i}}_{\alpha },t\right)$ and $\rho_{2}\left({\hat{i}}_{\beta },t\right)$ can be designed as:


$$\left\{ \begin{array}{c} \rho_{1}\left({\hat{i}}_{\alpha },t\right)=-\frac{R}{L_{s}}{\hat{i}}_{\alpha }+\frac{\text{1}}{L_{s}}u_{\alpha }\\ \rho_{2}\left({\hat{i}}_{\beta },t\right)=-\frac{R}{L_{s}}{\hat{i}}_{\beta }+\frac{\text{1}}{L_{s}}u_{\beta } \end{array}\right.$$ (16)

Obtained from (13):


{−RLsi^α+1Lsuα−δ1|i^α|1/12\nulldelimiterspace2≤0−RLsi^β+1Lsuβ−δ1|i^β|1/12\nulldelimiterspace2≤0 (17)

After selecting an appropriate $\delta_{1}>0$ from (17), the observer gains $k_{1}$ and $k_{2}$ must satisfy the constraints in (14) to ensure convergence performance.

The α and β axis current error state equations are obtained by subtracting (5) from (15):


ddt[i¯αi¯β]=−RLs[i¯αi¯β]+1Ls[eαeβ]−1Ls[k1|i¯α|1/12\nulldelimiterspace2sign(i¯α)+∫k2sign(i¯α)dtk1|i¯β|1/12\nulldelimiterspace2sign(i¯β)+∫k2sign(i¯β)dt] (18)

When the observer’s state variables reach the sliding surface ${\bar{i}}_{\alpha }={\bar{i}}_{\beta }=0$, the observer states will remain on the sliding surface thereafter. Based on the equivalent control principle, the observed back EMF can be expressed as:


[e^αe^β]=[eαeβ]eq=[k1|i¯α|1/12\nulldelimiterspace2sign(i¯α)+∫k2sign(i¯α)dtk1|i¯β|1/12\nulldelimiterspace2sign(i¯β)+∫k2sign(i¯β)dt] (19)

#### Switching function.

**Fig 3:**
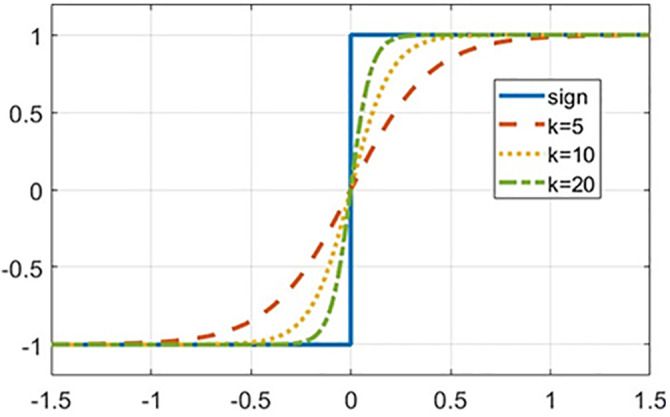
Convergence characteristics of the continuous function.

In traditional sliding mode observer design, the sign function is commonly used as the switching function. Due to the discontinuity of the sign function itself, it is easy to cause oscillations in the system during operation. In order to further reduce the effect of system chattering on control performance, the paper adopts a continuous function instead of the sign function sign, where the continuous function is represented as:


$$F(s)=\frac{e^{ks}-1}{e^{ks}+1}$$ (20)

k is a positive constant that determines the convergence characteristics of the function. When k takes different values, F(s) is shown in Fig 3. It can be seen that as k decreases, the curve changes more smoothly, and the system’s chattering effect decreases, but it will also reduce the speed at which the system approaches the sliding surface; The larger the value of k, the faster the convergence speed of the function, and the closer the effect is to the sign function, which can cause significant jitter. This article selects k=10 to balance the convergence speed of the system and suppress chattering.

At this point, the back EMF can be expressed as:


[e^αe^β]=[k1|i¯α|1/12\nulldelimiterspace2F(i¯α)+∫k2F(i¯α)dtk1|i¯β|1/12\nulldelimiterspace2F(i¯β)+∫k2F(i¯β)dt] (21)

### Model Predictive Control of PMSM Current

#### Single vector model predictive current control.

Traditional MPC consists of three key components: prediction model, receding horizon optimization, and feedback correction. At each sampling instant, the system predicts future outputs based on the established prediction model and given input information. These predicted outputs provide prior knowledge for the control system to evaluate a cost function over a defined time horizon, solving an optimization problem to determine the optimal control input through this continuous rolling optimization process. Finally, the actual system output is fed back to the controller for online error correction. For PMSM current control, FCS-MPC leverages the inherent discrete switching characteristics of inverters. It predicts future system states based on the controlled object’s mathematical model and a finite set of voltage vectors. FCS-MPC evaluates all possible switch states based on a predefined objective function and outputs the optimal voltage vector. This method has gained widespread attention in the field of motor control due to its fast dynamic response, non-linear, multi-objective, and multi constraint processing capabilities. For surface mounted PMSM, the prediction model is usually based on the current balance equation of dq rotating coordinate system:


$$\frac{d}{dt}\left[\begin{array}{c} i_{d}\\ i_{q} \end{array}\right]=\left[\begin{array}{c} -\frac{R}{L_{s}}i_{d}+\omega_{e}i_{q}+\frac{\text{1}}{L_{s}}u_{d}\\ -\frac{R}{L_{s}}i_{q}-\frac{\omega_{e}}{L_{s}}\psi_{f}-\omega_{e}i_{d}+\frac{\text{1}}{L_{s}}u_{q} \end{array}\right]$$ (22)

Discretize the continuous current equation using the forward Euler method:


$$\frac{di}{dt}=\frac{i_{k+1}-i_{k}}{T_{s}}$$ (23)

where $T_{s}$ is the current loop control period, representing the interval between two discrete moments.

When the current control cycle is sufficiently small, it can be assumed that parameters such as speed, inductance, and resistance remain essentially constant within this short period. Under these conditions, the dq -axis current values at the next time instant k+1 can be predicted using the current measurements of current, electrical angular velocity, and candidate voltage vectors. By combining (22) and (23), the discrete-time current prediction equations are obtained as follows:


$$\left[\begin{array}{c} i_{d}^{k+1}\\ i_{q}^{k+1} \end{array}\right]=\left[\begin{array}{c} i_{d}^{k}+\frac{T_{s}}{L_{s}}\left(u_{d}^{k}-Ri_{d}^{k}+\omega_{e}^{k}L_{s}i_{q}^{k}\right)\\ i_{q}^{k}+\frac{T_{s}}{L_{s}}\left(u_{q}^{k}-Ri_{q}^{k}-\omega_{e}^{k}\psi_{f}-\omega_{e}^{k}L_{s}i_{q}^{k}\right) \end{array}\right]$$ (24)

In model predictive control, the optimization is primarily achieved by constructing a cost function for online optimization, with the optimal voltage vector selected by this function being applied in the next sampling period. This study employs the differences between the predicted values ($i_{d}$, $i_{q}$) and their respective reference values as evaluation metrics. The optimal solution is obtained by comparing the cost functions generated under different voltage vectors’ effects on $i_{d}$ and $i_{q}$ feedback.

The candidate voltage vectors are input into the current prediction model to obtain their corresponding predicted current values. These values are then systematically evaluated through the objective cost function. For model predictive current control using dq axis currents as system variables, the cost function is constructed with squared error terms as follows:


$$J={\left(i_{d}^{ref}-i_{d}^{k+1}\right)}^{2}+{\left(i_{q}^{ref}-i_{q}^{k+1}\right)}^{2}$$ (25)

In the above equation, $i_{d}^{ref}$ and $i_{q}^{ref}$ represent the reference values for the stator currents. The closer the predicted values are to their reference values, the smaller the corresponding cost functionJ becomes. In MPCC, the d axis and q axis currents share the same physical dimensions and are assigned equal priority. Therefore, no weighting coefficients are applied between them in the cost function.

**Fig 4:**
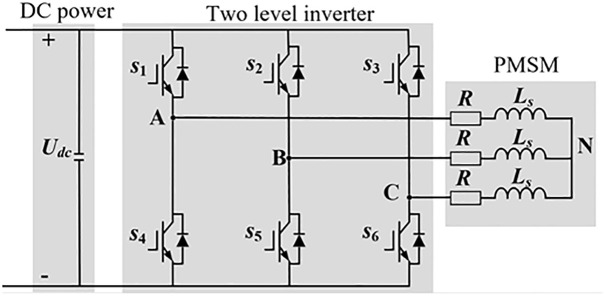
Three-phase two-level inverter.

The study employs a three-phase two-level voltage source inverter to power the PMSM, with its topological structure shown in Fig 4. Here, *s*_1_, *s*_2_, and *s*_3_ represent the switching signals for the upper-arm IGBT of the three phases in the two-level inverter, while *s*_4_, *s*_5_, and *s*_6_ correspond to the lower-arm IGBT switching signals. 1 indicates the IGBT is in the on state, and 0 denotes the off state.

**Fig 5:**
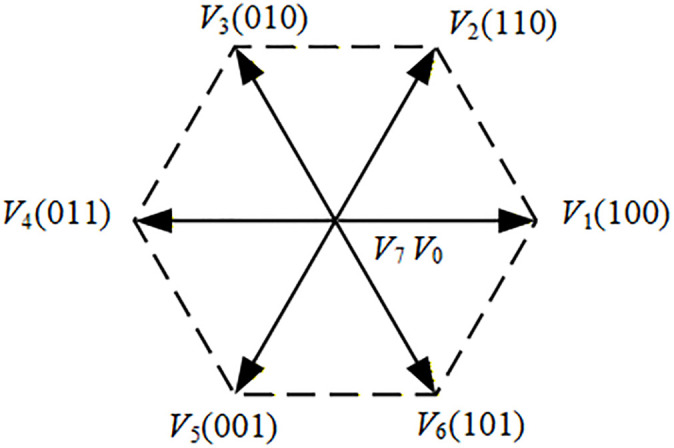
Basic voltage vectors of a three-phase two-level inverter.

Due to the complementary switching states of the upper and lower IGBT arms in the inverter, there exist a total of 8 possible switching combinations. Among these, 6 combinations are active switching states that establish electrical connection between the DC bus and the motor terminals. When these 6 active switching combinations are converted into space vector form, they yield 6 voltage space vectors with fixed magnitudes and spatial orientations, known as active voltage vectors. Additionally, there are 2 switching states that disconnect the DC bus from the motor side, occurring when either all upper-arm or all lower-arm IGBT are simultaneously turned on or off. These 2 switching combinations correspond to zero voltage vectors in space vector representation. This results in 7 distinct voltage vectors (6 active vectors and 2 zero vectors), as illustrated in Fig 5.

Based on coordinate transformation theory, the current equations for the d axis and q axis in the {dq} reference frame can be derived, enabling the implementation of model predictive current control. The model predictive control algorithm selects the optimal control input exclusively from the 6 active vectors and 2 zero vectors, it is termed finite control set model predictive control (FCS-MPC).

#### Dual-vector model predictive current control.

The conventional FCS-MPCC strategy for PMSM applies only one basic voltage vector per control cycle. Typically, the predicted current values after applying the selected voltage vector will either be lower or higher than the reference values, making it impossible to achieve deadbeat control of d and q axis currents. This results in significant ripple in the controlled variables $i_{d}$ and $i_{q}$, leading to poor steady-state performance of the control system. To address the unsatisfactory steady-state characteristics of FCS-MPCC for PMSM, some researchers have attempted to introduce duty cycle modulation into traditional MPCC. This approach adjusts the ratio between active vectors and zero vectors’ application time within one control cycle, thereby enabling the model predictive current control to regulate the output vector magnitude and consequently suppress undesirable current fluctuations.

Through the deadbeat control method for q axis current, the duty cycle calculation for the sampling period is realized. Specifically, this ensures that the $i_{q}$ value at k+1 moment equals $i_{q}^{ref}$, expressed as:


$$i_{q}^{k+1}=i_{q}^{k}+s_{opt}T_{s}\gamma_{opt}+s_{0}\left(T_{s}-T_{s}\gamma_{opt}\right)=i_{q}^{ref}$$ (26)

It is obtained:


$$\gamma_{opt}=\frac{i_{q}^{ref}-i_{q}^{k}-s_{0}T_{s}}{T_{s}\left(s_{opt}-s_{0}\right)}$$ (27)

where $T_{s}$ represents the sampling period, $s_{opt}$ and $\gamma_{opt}$ denote the slope and duty cycle of $i_{q}$ when the optimal voltage vector is applied, $s_{0}$ is the slope of $i_{q}$ under zero voltage vector application. From (22):


$$\begin{array}{c} s_{0}={\frac{di_{q}}{dt}|}_{u_{q}=0}=-\frac{\text{1}}{L_{s}}\left(Ri_{q}+\omega_{e}L_{s}i_{d}+\omega_{e}\psi_{f}\right)\\ s_{opt}={\frac{di_{q}}{dt}|}_{u_{q}=u_{q\_opt}}=s_{0}+\frac{u_{q\_opt}}{L_{s}} \end{array}$$ (28)

where $u_{q\_opt}$ is the q axis component of the optimal voltage vector.

By synthesizing a new voltage vector from active vectors and zero vectors, the system can achieve more precise tracking of the reference voltage, thereby improving steady-state performance. Since the zero vector is excluded from the candidate set for optimal voltage selection in this strategy, the selection is confined to the 6 active voltage vectors. Building upon the duty cycle MPCC approach, the dual vector MPCC (DVMPCC) strategy extends the selection range of the second vector from just zero vectors to all available inverter voltage vectors. This advanced strategy enables the selection of two distinct voltage vectors within a single sampling period. By optimally allocating their respective application times, a new synthesized voltage vector is generated that more closely approximates the target vector. Unlike conventional single vector MPCC, where output vectors are limited to fixed directions and magnitudes, this method offers infinite possible time-based combinations. Consequently, the synthesized voltage vectors are no longer constrained to a few discrete orientations and amplitudes. This enhanced flexibility minimizes the discrepancy between predicted and reference current values, significantly improving the system’s steady-state performance. To determine the optimal vector combination, a rational time allocation between the two selected vectors must be implemented. The specific allocation method follows the q axis current deadbeat principle used in duty cycle control, expressed as:


$$i_{q}^{k+1}=i_{q}^{k}+s_{opt1}t_{opt1}+s_{i}\left(T_{s}-t_{opt1}\right)=i_{q}^{ref}$$ (29)

where $s_{opt1}$ and $s_{i}$ are the slopes of $i_{q}$ when the first and second optimal voltage vectors are applied, respectively. The first optimal voltage vector action time:


$$t_{opt1}=\frac{i_{q}^{ref}-i_{q}^{k}-s_{i}T_{s}}{s_{opt1}-s_{i}}$$ (30)

$s_{opt1}$ and $s_{i}$ can be represented as:


$$\begin{array}{c} s_{opt1}={\frac{di_{q}}{dt}|}_{u_{q}=u_{q\_opt1}}=s_{0}+\frac{u_{q\_opt1}}{L_{s}}\\ s_{i}={\frac{di_{q}}{dt}|}_{u_{q}=u_{q\_i}}=s_{0}+\frac{u_{q\_i}}{L_{s}} \end{array}$$ (31)

where $u_{q\_i}$ is the q axis component of the second optimal voltage vector.

#### Numerical calculation.

**Fig 6:**
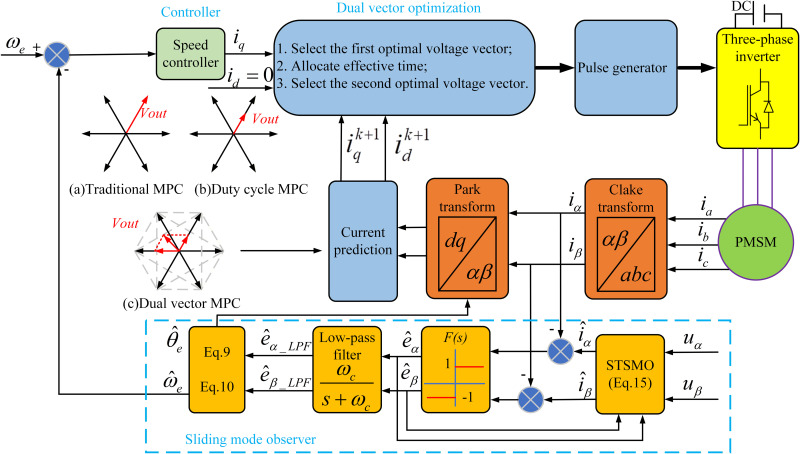
DVMPCC block diagram.

The PMSM current finite set model predictive control based on the super twisting sliding mode observer proposed in this article is shown in Fig 6.

On the basis of selecting the optimal voltage vector in the traditional MPCC strategy, another voltage vector selection is carried out, and then the action time is calculated and allocated to combine the two voltage vectors into a new voltage vector. Finally, the value function is compared one by one to obtain the optimal second voltage vector and the allocation scheme of action time, thereby improving the steady-state performance of the control system. Among them, the two voltage vectors before and after are selected from the 8 basic voltage vectors of the inverter. Figs 6(a), 6(b), and 6(c) show the voltage vector selection ranges for three control methods: traditional MPC, duty cycle MPC, and DVMPC. Traditional MPC can only select the optimal vector from seven voltage vectors, with a fixed direction and size; The duty cycle MPC voltage vector has adjustable magnitude and fixed direction, which is the synthesis of the optimal voltage vector and zero vector; Dual vector MPC combines the optimal voltage vector with any voltage vector, and the direction and amplitude of the output voltage vector can be adjusted. To verify the effectiveness of the designed scheme, numerical calculations and result analysis will be conducted in this section.

### STSMO performance analysis

**Fig 7:**
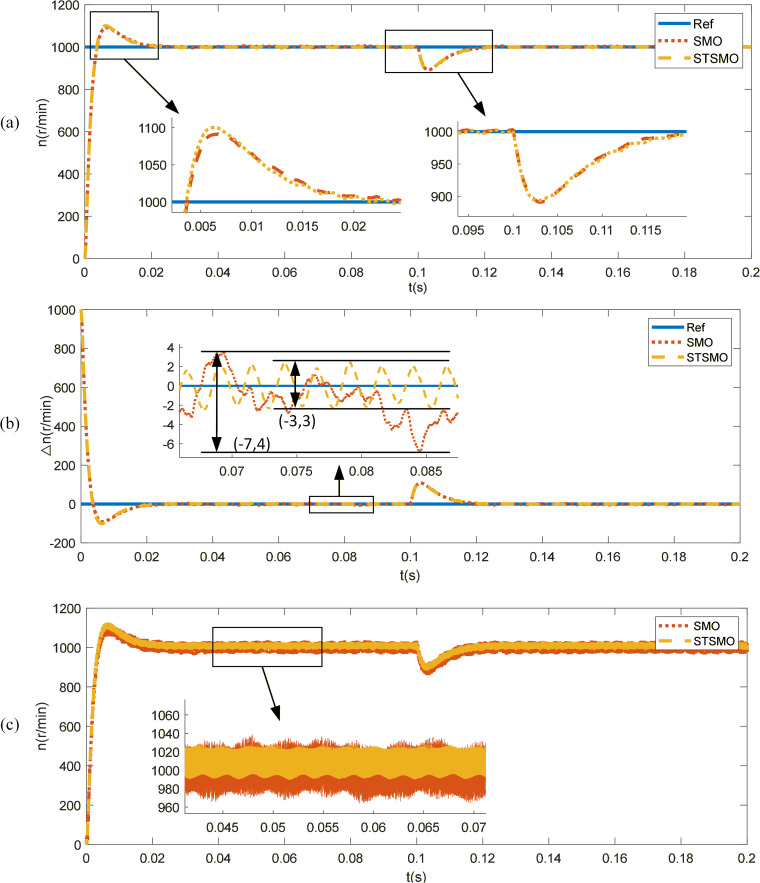
Response of SMO and STSMO. (a) Speed response of SMO and STSMO. (b) Speed error. (c) Observed rotational speed.

To study the effect of PMSM using STSMO, a reference speed of 1000r/min is given, and a sudden load of 1Nm is applied at t = 0.1s. The control effects of STSMO and traditional SMO (without phase and amplitude compensation) are shown in Fig 7. As shown in the Fig, both sliding mode observers can quickly track the reference speed. The traditional SMO observer has a peak speed of 1092r/min during the dynamic response phase, with an overshoot of 9.2% and a peak time of 0.007s. After entering the steady-state response phase, the maximum speed error is 7r/min, and the speed oscillates between 996r/min and 1007r/min; The STSMO observer has a peak speed of 1100r/min during the dynamic response phase, with an overshoot of 10% and a peak time of 0.0063s. After entering the steady-state response phase, the maximum speed error is 3r/min, and the speed oscillates between 997r/min and 1003r/min. After a sudden increase in load, both methods are able to quickly track the actual speed again. The SMO and STSMO speeds dropped sharply to 891r/min and 890r/min, respectively, and quickly returned to the reference speed under controller adjustment. As shown in Fig 7 (c), the observed speed fluctuation of the STSMO observer is smaller than that of the SMO observer.

**Fig 8:**
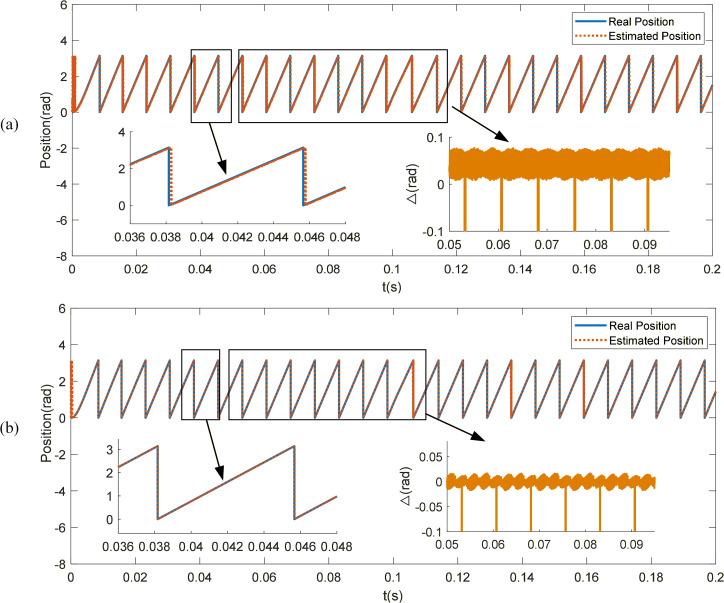
Position tracking curve. (a) SMO rotor position. (b) STSMO rotor position.

Fig 8 illustrates the position tracking performance of the two control methods. It can be observed that due to the lack of phase and amplitude compensation, the traditional SMO, although generally consistent with the actual angle, introduces a noticeable phase delay when low-pass filtering is applied to the equivalent control signal. After compensation, the STSMO provides highly accurate rotor position observation. The predicted angle closely aligns with the actual angle, with the error maintained within 0.02 rad. Compared to the traditional SMO, the STSMO exhibits a smaller position error and continues to accurately and rapidly track the position even after a sudden load is applied.

In summary, the calculation results demonstrate that the optimized STSMO achieves more accurate rotor position estimation compared to the traditional SMO. It provides smoother speed response, effectively reduces system chattering, minimizes speed fluctuations, enhances system stability, and delivers superior speed tracking performance under load conditions.

### STSMO-DVMPCC performance analysis

**Fig 9:**
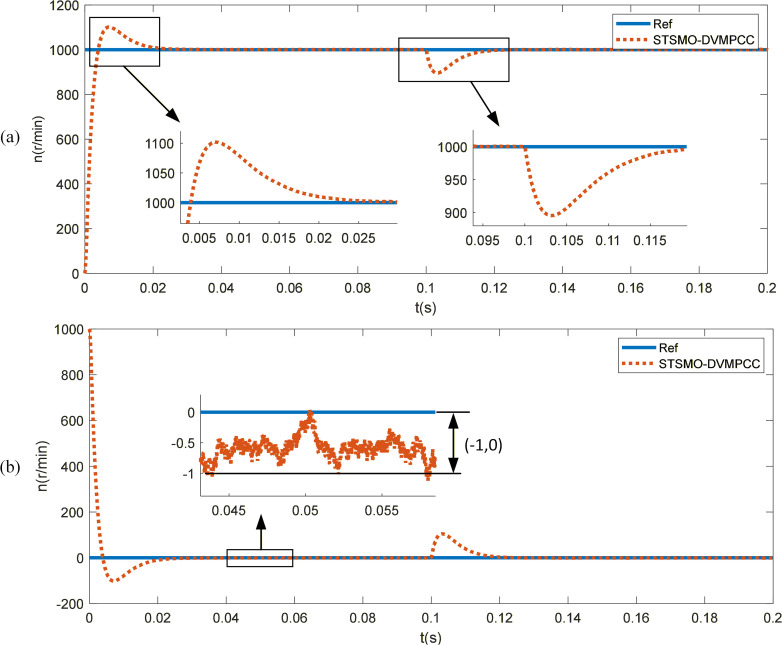
Speed response of STSMO-DVMPCC. (a) Speed response curve of STSMO-DVMPCC. (b) Speed error curve.

The dual vector model predictive current control improves the motor’s dynamic performance and steady-state accuracy by optimizing the selection of voltage vectors and the allocation of their application time. Fig 9 shows the speed tracking performance of PMSM with the optimized STSMO-based DVMPCC. As can be seen from the Fig, when responding to a step reference speed (1000 r/min), the STSMO-DVMPCC reaches a peak speed of 1102 r/min, indicating an overshoot of 10.2%, with a peak time of 0.007 s. During steady-state operation, the maximum speed error is 1 r/min, with speed oscillations between 1000–1001 r/min. When a sudden load is applied, the speed drops to 895 r/min but quickly recovers to track the reference value.

**Fig 10:**
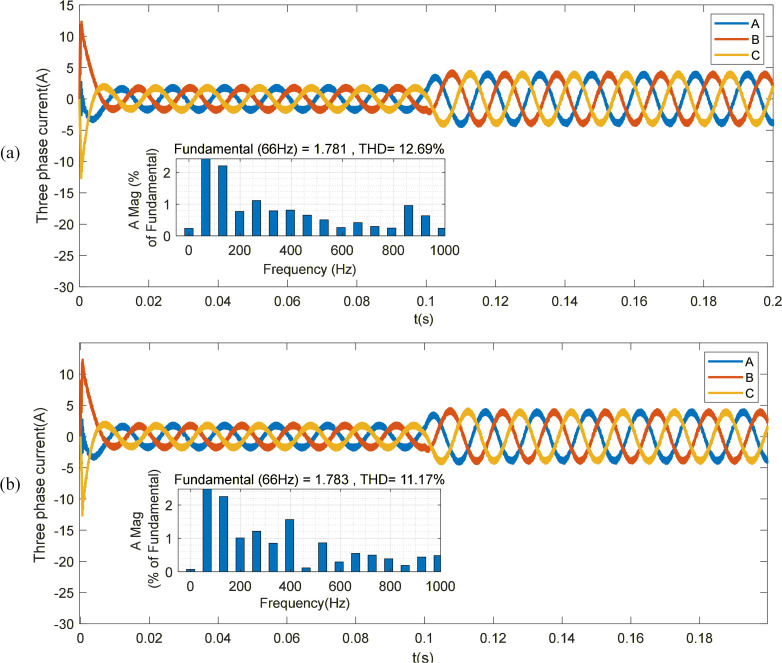
PMSM stator current. (a) Stator current under STSMO-MPCC control. (b) Stator current under STSMO-DVMPCC control.

As Fig 10 shows, compared with STSMO-MPCC control, the three-phase stator current waveforms under STSMO-DVMPCC control exhibit smoother profiles and reduced current ripple. Under steady-state operating conditions, the total harmonic distortion (THD) of phase A current decreases from 12.69% to 11.17%, indicating a more stable motor response process.

## Conclusion

To enhance the sensorless control performance of PMSM, this study improves upon the traditional SMO by introducing an optimized STSMO, effectively mitigating system chattering. The accuracy of rotor position estimation is enhanced via comprehensive phase and amplitude compensation of the observed signals. Furthermore, chattering is further suppressed by substituting the sign function with a continuous sigmoid function. The optimized STSMO exhibits superior tracking performance and contributes to smoother motor operation. For current loop regulation, a DVMPC strategy is employed in place of conventional PI control, significantly improving dynamic response. This method effectively overcomes the inherent trade-off in PI control between dynamic and steady-state performance over wide speed ranges. In comparison with standard MPC, the proposed approach notably reduces current harmonics. Simulation and experimental results confirm that the combined STSMO-DVMPCC strategy offers an effective sensorless control solution for PMSM applications.
